# A High-Density Consensus Map of Common Wheat Integrating Four Mapping Populations Scanned by the 90K SNP Array

**DOI:** 10.3389/fpls.2017.01389

**Published:** 2017-08-09

**Authors:** Weie Wen, Zhonghu He, Fengmei Gao, Jindong Liu, Hui Jin, Shengnan Zhai, Yanying Qu, Xianchun Xia

**Affiliations:** ^1^College of Agronomy, Xinjiang Agricultural University Urumqi, China; ^2^National Wheat Improvement Center, Institute of Crop Science, Chinese Academy of Agricultural Sciences Beijing, China; ^3^International Maize and Wheat Improvement Center (CIMMYT) Beijing, China; ^4^Crop Breeding Institute, Heilongjiang Academy of Agricultural Sciences Harbin, China

**Keywords:** linkage map construction, map integration, SNP assay, Spearman rank correlation coefficient, *Triticum aestivum*

## Abstract

A high-density consensus map is a powerful tool for gene mapping, cloning and molecular marker-assisted selection in wheat breeding. The objective of this study was to construct a high-density, single nucleotide polymorphism (SNP)-based consensus map of common wheat (*Triticum aestivum* L.) by integrating genetic maps from four recombinant inbred line populations. The populations were each genotyped using the wheat 90K Infinium iSelect SNP assay. A total of 29,692 SNP markers were mapped on 21 linkage groups corresponding to 21 hexaploid wheat chromosomes, covering 2,906.86 cM, with an overall marker density of 10.21 markers/cM. Compared with the previous maps based on the wheat 90K SNP chip detected 22,736 (76.6%) of the SNPs with consistent chromosomal locations, whereas 1,974 (6.7%) showed different chromosomal locations, and 4,982 (16.8%) were newly mapped. Alignment of the present consensus map and the wheat expressed sequence tags (ESTs) Chromosome Bin Map enabled assignment of 1,221 SNP markers to specific chromosome bins and 819 ESTs were integrated into the consensus map. The marker orders of the consensus map were validated based on physical positions on the wheat genome with Spearman rank correlation coefficients ranging from 0.69 (4D) to 0.97 (1A, 4B, 5B, and 6A), and were also confirmed by comparison with genetic position on the previously 40K SNP consensus map with Spearman rank correlation coefficients ranging from 0.84 (6D) to 0.99 (6A). Chromosomal rearrangements reported previously were confirmed in the present consensus map and new putative rearrangements were identified. In addition, an integrated consensus map was developed through the combination of five published maps with ours, containing 52,607 molecular markers. The consensus map described here provided a high-density SNP marker map and a reliable order of SNPs, representing a step forward in mapping and validation of chromosomal locations of SNPs on the wheat 90K array. Moreover, it can be used as a reference for quantitative trait loci (QTL) mapping to facilitate exploitation of genes and QTL in wheat breeding.

## Introduction

Common wheat (*Triticum aestivum* L.) is an allohexaploid species (*2n* = *6x* = 42, AABBDD) derived from the hybridization of diploid *Aegilops tauschii* (DD) and tetraploid wild emmer (AABB) 10,000 years ago (Dubcovsky and Dvorak, 2007), with a genome size of about 17 gigabase (Gb) (Choulet et al., 2014); it is one of the most important crops, supplying food for 35% of the world population (International Wheat Genome Sequencing Consortium, 2014). Intense breeding activities over the past century to improve wheat varieties have been carried out to meet demand from a gradually increasing human population. Breeding is a long-term process and molecular marker tools open the way for more rapid and efficient breeding strategies (Tester and Langridge, 2010). Over the last three decades molecular markers have increasingly served as a tool for genetic analysis of plant species. Decreasing costs of marker assays are making marker-assisted selection (MAS) increasingly used in breeding programs (Soto-Cerda et al., 2015; Vinod et al., 2015; Yaniv et al., 2015; Arruda et al., 2016; Vasistha et al., 2016). Linkage mapping is a major strategy to identify markers tightly linked with quantitative trait loci (QTL) underlying many economically important traits (Zheng et al., 2015; Dong et al., 2016; Su et al., 2016).

Genetic linkage maps play an important role in genomic studies, including QTL mapping, MAS and map-based cloning. Genetic maps of common wheat have progressed with the development of different types of DNA markers, from restriction fragment length polymorphisms (RFLP) (Chao et al., 1989), through random amplified polymorphic DNA (RAPD) markers (Joshi and Nguyen, 1993), amplified fragment length polymorphisms (AFLPs) (Hartl et al., 1999) and simple sequence repeats (SSRs) (Röder et al., 1998), to high-throughput marker systems recently used in wheat. This last group includes diversity array technology (DArT) (Semagn et al., 2006), genotyping-by-sequencing (GBS) (Li et al., 2015) and single nucleotide polymorphisms (SNPs) (Cabral et al., 2014). Liu et al. (2016) constructed three high-density SNP based maps containing 5,950, 4,861, and 8,376 markers, respectively. Gardner et al. (2016) constructed a SNP map using an eight-parent MAGIC population, comprising 18,601 SNP markers, Zhai et al. (2016) constructed a high-density map with 10,816 SNP and 174 SSR markers to identify tightly linked markers with important QTLs, Assanga et al. (2017) found eight tightly linked SNPs with *Wsm2* gene by a dense SNP map with a total length of 2,079.5 cM.

The use of a linkage map based on a single mapping population is often limited by the genetic background and a low level of polymorphism (Galeano et al., 2011), whereas a consensus map combining genetic information from multiple populations with different genetic backgrounds offer opportunities to map more markers and provide greater genome coverage with higher marker densities. They also validate marker order, and identify chromosomal rearrangements (Salvi et al., 2009; Maccaferri et al., 2015), for example, the cyclic translocation involving chromosomes 4A, 5A, and 7B (Devos et al., 1995). A consensus map with high-density markers can be used to scan the whole genome to identify different kinds of chromosomal rearrangements such as translocations, inversions, and duplications (Marone et al., 2012).

The several consensus maps include the map for common wheat based on SSR markers (Somers et al., 2004), and that for durum based on SSR and DArT markers (Marone et al., 2012). High-throughput sequencing technology provides millions of SNP markers (Kumar et al., 2012; Winfield et al., 2016). Several SNP-based consensus wheat maps have been developed in wheat (Cavanagh et al., 2013; Wang et al., 2014; Yu and Barbier, 2014; Li and Wang, 2015; Maccaferri et al., 2015).

The wheat 90K Infinium iSelect SNP array is a currently useful genetic tool for wheat research (Avni et al., 2014; Colasuonno et al., 2014; Russo et al., 2014; Sukumaran et al., 2015). Wang et al. (2014) constructed a consensus map using six mapping populations and mapped 40,267 SNPs (hereafter, 40K) from the 81,587 on the wheat 90K array; however, more than half were not mapped. The objective of the present study was to develop a high-density consensus map using four recombinant inbred line (RIL) populations to validate the chromosomal locations of mapped SNPs, and to increase the marker density in the map as a reference resource for genetic studies and molecular breeding. In addition, the collinearity of markers between homoeologous chromosomes and chromosomal rearrangements were revealed by marker duplication across chromosomes. The present high-density consensus map will be helpful in mapping QTLs for important traits and developing high-throughput markers for MAS in wheat breeding.

## Materials and Methods

### Mapping Populations

Four RIL populations derived from crosses Doumai × Shi 4185 (275 *F*_2:6_ RILs), Gaocheng 8901 × Zhoumai 16 (176 *F*_2:6_ RILs), Linmai 2 × Zhong 892 (273 *F*_2:6_ RILs) and Zhou 8425B × Chinese Spring (245 *F*_2:8_ RILs) were used for consensus map construction (**Table 1**). Doumai is a Chinese winter wheat line with a large grain size, while Shi 4185 is a winter wheat cultivar widely grown in Hebei province (Pedigree: Zhi 8094/Baofeng 7228//Shi 84-7120); Gaocheng 8901 is a winter wheat cultivar grown in Hebei province with good processing quality (Pedigree: 77546/Linzhangmai) and Zhoumai 16 is a widely grown facultative wheat cultivar in Henan province (Pedigree: Zhoumai 9/Zhou 8425B); Linmai 2 is a winter wheat cultivar grown in Shanxi province (Pedigree: Lumai 23/Lin 90-15), while Zhong 892 is a winter wheat line with high resistance to black point disease; Zhoumai 8425B is an elite facultative wheat line widely used as a parent in Chinese wheat breeding programs (Pedigree: Zhou 78A/Annong 7959), and Chinese Spring is a Chinese landrace wheat widely used in genetic studies in the world.

**Table 1:** Summary of four individual maps used to construct the consensus map.

Cross	Type	Size	Sub genome	Markers	Bins	Linkage groups	Total length (cM)	Marker density (markers/cM)
Doumai × Shi 4185	*F*_2:6_	275	A	4,538	1,243	7	839.70	5.40
			B	5,041	1,259	7	770.64	6.54
			D	1,407	338	7	545.73	2.58
			Total	10,986	2,840	21	2,156.07	5.10
Gaocheng 8901 × Zhoumai 16	*F*_2:6_	176	A	4,574	1,282	10	1,187.30	3.85
			B	5,911	1,607	7	1,386.09	4.26
			D	1,334	353	14	587.75	2.27
			Total	11,819	3,242	31	3,161.14	3.74
Linmai 2 × Zhong 892	*F*_2:6_	273	A	3,754	1,309	12	1,252.64	3.00
			B	5,319	1,622	22	1,355.18	3.92
			D	751	267	11	347.00	2.16
			Total	9,824	3,198	45	2,954.82	3.32
Zhou 8425B × Chinese Spring	*F*_2:8_	245	A	5,965	1,502	7	847.13	7.04
			B	7,699	1,606	7	895.75	8.60
			D	1,198	352	7	547.18	2.19
			Total	14,862	3,460	21	2,290.06	6.49

### DNA Extraction and Genotyping

DNA was extracted from leaves of 15-day-old seedlings following the cetyltrimethyl ammonium bromide (CTAB) protocol (Sharp et al., 1988); 1 μl of extracted DNA solution was used to check the DNA quality by electrophoresis in a 1% agarose gel, and the DNA concentration was measured by NanoDrop 2000C (Thermo Scientific, United States).

The populations were genotyped using 90K wheat Infinium iSelect SNP arrays developed by Illumina Inc.^1^ SNP assays were performed at CapitalBio Ltd.^2^ in Beijing. Genotype calling was performed using a combination of GenomeStudio v2011.1 and GenomeStudio Polyploid Clustering V1.0 as described previously (Wang et al., 2014). The quality of genotypic data was controlled according to the following rules: (i) data points showing heterozygosity were considered to be missing data, (ii) SNPs with missing data over 10% were filtered, and (iii) allelic frequencies at a locus should be between 0.3 and 0.7.

### Single Map Analysis

The linkage map for each population was constructed in four steps; firstly, SNP markers, among which the recombination frequency was estimated as zero, were placed into a recombinant bin using the BIN function in the QTL IciMapping V4.0 software (Li et al., 2007), and the marker with the minimum percentage of missing data among the RILs was selected to represent the recombinant bin as a frame marker, or randomly selected in cases where more than one marker had the same minimum percentage of missing data; secondly, frame markers were sorted into groups using the “Grouping” function in JoinMap 4.0 (Stam, 1993) with LOD thresholds ≥ 7; thirdly, the frame markers in identified groups were ordered and genetic distances were calculated using the “AutoMap” command in MapDisto 1.7 (Lorieux, 2012) and binned markers from the first step were reintegrated into linkage groups based on the frame markers; finally, the assignment and orientation of linkage groups to chromosomes were based on the best blast hits from the alignment of the SNP-flanking sequences with the chromosome sequence survey (CSS) database^3^.

### Construction of the Consensus Map

The consensus map was constructed using MergeMap (Wu et al., 2008b), taking into account the individual maps to calculate the consensus marker orders of linkage groups. First, each linkage group was converted to directed acyclic graphs (DAGs) (Yap et al., 2003), and then, a consensus graph was merged based on shared vertices, and finally, each consensus DAG was linearized using a mean distance approximation. Equal weights (1.0) were assigned to each linkage group specified in the configuration files. To correct the inflation of genetic distances between markers in the consensus map (Close et al., 2009), we rescaled the consensus map positions for each chromosome by the slope of the consensus map positions regressed on individual map positions following Cavanagh et al. (2013). The linkage groups were assigned to the chromosomes according to putative physical positions (obtained by blasting the SNP-flanking sequences against the CSS sequences) of the majority of SNP markers. The SNP markers that had the same genetic positions on the consensus map were assigned into a same recombinant bin. The degree of correspondence of marker order between individual and consensus maps was evaluated by pairwise Spearman rank correlation coefficients using SAS 9.4.

To enrich the number of molecular markers on the hexaploid wheat genetic map, an integrated consensus map was constructed with the data of the present study and those from five other reports (Cavanagh et al., 2013; Cabral et al., 2014; Wang et al., 2014; Gardner et al., 2016; Zhai et al., 2016) using MergeMap (Wu et al., 2008b).

### Integration of the Consensus and Wheat Expressed Sequence Tag (EST) Chromosome Bin Maps

Flanking sequences of SNPs mapped in the consensus map were used to blastn the 6,596 mapped wheat expressed sequence tags (ESTs)^4^ on the wheat Chromosome Bin Map (Qi et al., 2004), with an *E*-value < 1*e* - 10. After filtering the redundant ESTs in a chromosomal location, SNP-BIN relationships were established considering the relationships between SNP-EST and EST-BIN. The percentages of Chromosome Bin related to SNP markers relative to the 159 Bins over the genome (Qi et al., 2004) were estimated to describe the fraction of coverage of the consensus map against the wheat genome (Lazo et al., 2004).

### Map Validation

To confirm the marker order in the present consensus map, marker assignments to linkage group were compared with the corresponding positions in the consensus map constructed by Wang et al. (2014) and the putative physical positions in the wheat genome; the putative physical positions of markers were obtained by blastn the SNP-flanking sequences against the IWGSC1+popseq database^5^ (Choulet et al., 2014; International Wheat Genome Sequencing Consortium, 2014; Chapman et al., 2015), with a filtering threshold of *E*-value < 1*e* - 10. For multiple physical positions corresponding to a specific marker, the position matching the lowest *E*-value was used to represent the physical locations of markers. Spearman rank correlation coefficients were calculated to evaluate degree fit between physical and genetic positions of SNP markers on the consensus map, and also the marker order consistency between our consensus map and the map constructed by Wang et al. (2014). The quality of the integrated consensus map was evaluated in comparison with a consensus map of tetraploid wheat (Maccaferri et al., 2015) and the map constructed by Wang et al. (2014).

## Results

### Characterization of the Individual Maps

After filtering the genotypic data based on the criteria mentioned above, 11,012 (Doumai × Shi 4185), 11,979 (Gaocheng 8901 × Zhoumai 16), 10,443 (Linmai 2 × Zhong 892), and 14,955 (Zhou 8425B × Chinese Spring) markers were used to construct the genetic linkage maps.

For the Doumai × Shi 4185 population, 11,012 polymorphic markers were assigned to 2,851 recombinant bins, represented by 2,851 frame marker, of which 2,840 were grouped into 21 linkage groups corresponding to the 21 wheat chromosomes, whereas 11 were not linked with any other frame marker. Finally, 10,986 polymorphic markers covering 2,156.07 cM were mapped, with an average marker density of 5.1 markers/cM (**Table 1** and Supplementary Table S1). The D genome contained the least markers (1,407, 12.8%) compared to the A (4,538, 41.3%) and B (5,041, 45.9%) genomes.

For the Gaocheng 8901 × Zhoumai 16 population, 11,979 polymorphic markers were mapped based on 3,284 frame markers, of which 3,242 were grouped into 31 linkage groups covering all wheat chromosomes, and 42 were not linked to any frame markers. Due to large genetic distances among some markers, seven chromosomes were split into multiple linkage groups (1A, 2D, 3D, 4D, 5A, 5D, and 6D corresponding to 3, 2, 2, 2, 2, 4, and 2 groups, respectively), and the other 14 chromosomes each consisted of a single linkage group. Finally, 11,819 polymorphic markers were mapped, covering a total length of 3,161.14 cM with an average marker density of 3.74 markers/cM (**Table 1** and Supplementary Table S1). The D genome had the least markers (1,334, 11.3%) compared with the A (4,574, 38.7%) and B (5,911, 50%) genomes.

For the Linmai 2 × Zhong 892 population, 10,443 polymorphic markers were mapped based on 3,489 frame markers, of which 3,198 were grouped into 45 linkage groups covering all wheat chromosomes, 180 markers lacked linkage and 111 in three groups were not assigned to any chromosome. Because of the unbalanced distribution of markers on chromosomes, 11 chromosomes were split into multiple linkage groups (1A, 2A, 2B, 2D, 3B, 4B, 4D, 6A, 6B, 7B, and 7D each with 2, 3, 3, 2, 6, 2, 2, 3, 4, 5, and 3 groups, respectively), and the other 10 chromosomes each corresponded to single linkage groups. Finally, 9,824 polymorphic markers were mapped, covering a total length of 2,954.82 cM, with an average marker density of 3.32 markers/cM (**Table 1** and Supplementary Table S1). The D genome had the least markers (751, 7.6%) compared with the A (3,754, 38.2%) and B (5,319, 54.1%) genomes.

For the Zhou 8425B × Chinese Spring population, 14,955 polymorphic markers were represented by 3,498 frame markers, of which 3,460 were grouped into 21 linkage groups corresponding to the 21 wheat chromosomes, and 38 were unlinked to any groups. Finally, 14,862 markers mapped to 21 linkage groups covered 2,290.06 cM, with an average marker density of 6.49 markers/cM (**Table 1** and Supplementary Table S1). The D genome had the least markers (1,198, 8.1%) compared to the A (5,965, 40.1%) and B (7,699, 51.8%) genomes.

**FIGURE 1:**
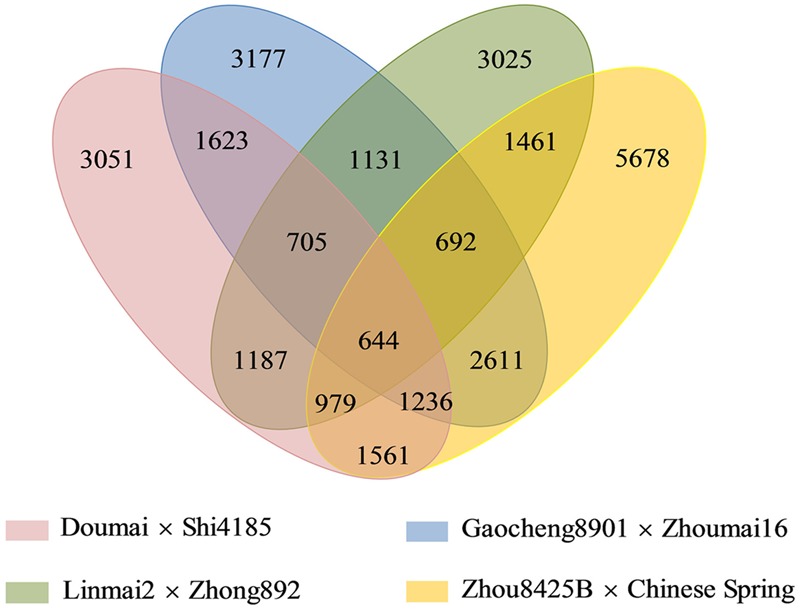
A four-way Venn diagram demonstrates all sets of common SNP markers among the four mapping populations.

Among 28,761 redundant markers on the four maps, 14,931 (51.9%) were mapped only in individual maps, whereas 9,574 (33.3%), 3,612 (12.6%), and 644 (2.2%) were shared by two, three and four individual maps, respectively (**Figure 1**). The percentages of markers mapped on single genetic maps ranged from 26.9% (Gaocheng 8901 × Zhoumai 16) to 38.2% (Zhou 8425B × Chinese Spring) of the total.

### Construction of the Consensus Map

The consensus map comprised 29,692 SNP markers belonging to 8,960 recombinant bins. All markers were assigned to 21 linkage groups corresponding to the 21 wheat chromosomes. The total length of the consensus map was 2,906.86 cM, with an average chromosome length of 138.42 cM, ranging from 67.71 cM (4D) to 288.3 cM (2B). The number of markers in each chromosome varied from 118 (4D) to 2,872 (2B), with an average of 1,414 markers per chromosome. The overall marker density was 10.21 markers/cM, ranging from 1.74/cM on 4D to 19.13/cM on 1B (**Table 2** and Supplementary Table S2).

**Table 2:** Characteristics of the consensus map.

Class	Markers	Recombination bins	Length (cM)	Marker density (markers/cM)
Chromosome				
1A	1,496	534	127.38	11.74
1B	2,282	470	119.27	19.13
1D	774	170	94.50	8.19
2A	1,885	549	152.96	12.32
2B	2,872	822	288.30	9.96
2D	728	194	100.64	7.23
3A	1,437	476	177.17	8.11
3B	2,261	702	149.72	15.10
3D	410	118	102.44	4.00
4A	1,547	468	165.54	9.35
4B	865	342	90.15	9.60
4D	118	75	67.71	1.74
5A	1,371	537	179.22	7.65
5B	2,741	763	198.74	13.79
5D	391	157	118.67	3.29
6A	1,953	455	144.96	13.47
6B	2,022	623	120.72	16.75
6D	388	136	94.52	4.10
7A	2,010	686	194.48	10.34
7B	1,835	556	110.32	16.63
7D	306	127	109.45	2.80
Genome				
A	11,699	3,705	1,141.71	10.25
B	14,878	4,278	1,077.22	13.81
D	3,115	977	687.93	4.53
Homoeologous group				
1	4,552	1,174	341.15	13.34
2	5,485	1,565	541.90	10.12
3	4,108	1,296	429.33	9.57
4	2,530	885	323.40	7.82
5	4,503	1,457	496.63	9.07
6	4,363	1,214	360.20	12.11
7	4,151	1,369	414.25	10.02
Total	29,692	8,960	2,906.86	10.21

A total of 11,699 (39.4%) markers were mapped to the A genome spanning 1,141.71 cM, with an average marker density of 10.25 markers/cM; 14,878 (50.1%) markers were located to the B genome covering 1,077.22 cM with a mean marker density of 13.81 markers/cM, and 3,115 (10.5%) markers were mapped to the D genome with a total length of 687.93 cM and an average marker density of 4.53 markers/cM.

When classified by homoeologous groups, the number of markers ranged from 2,530 (8.5%) in group 4 to 5,485 (18.5%) in group 2, with an average of 4,242; and total length varied from 323.4 cM (11.1%) in group 4 to 541.9 cM (18.6%) in group 2, with an average 415.27 cM. Homoeologous group 4 had the lowest density of 7.82 markers/cM and group 1 had the highest density of 13.34 markers/cM.

Comparison between the flanking sequences of SNP markers and the wheat genome CSS v2 database assigned 26,607 markers onto specific chromosome arms (Supplementary Table S2). The short and long arms were clearly separated for most chromosomes, except 2A and 7A (Supplementary Figure S1). The sequencing strategy of chromosome 3B (Choulet et al., 2014) did not distinguish the arms, thus, the SNP markers on 3B were not anchored to the specific chromosome arms.

Markers and recombinant bins were unevenly distributed along the chromosomes based on 5 cM genetic intervals (Supplementary Figure S2). The distribution of recombinant bins was quite consistent with the distribution of markers in most chromosomes with an average Person correlation coefficient of 0.65; exceptions included chromosomes 1B, 1D, 2D, 3B, 3D, and 5B, with an average Person correlation coefficient of 0.33. Although there were no large gaps (over 10 cM) on any chromosome, marker clustering occurred in some chromosomes (e.g., 2B, 3A, 4D, 6D, 7A, and 7D).

The degree of marker order consistency between individual maps and the consensus map was quite good; of 84 pairwise chromosome comparisons, 82 showed the Spearman rank correlation coefficients higher than 0.9, and only those for chromosomes 5D and 6D between cross Gaocheng 8901 × Zhoumai 16 and the consensus map were less than 0.9 (**Table 3**).

**Table 3:** Spearman rank correlation coefficients of marker positions in linkage groups between the individual maps and the consensus map.

Chromosome	Doumai × Shi 4185	Gaocheng 8901 × Zhoumai 16	Linmai 2 × Zhong 892	Zhou 8425B × Chinese Spring
1A	1.00	0.96	1.00	1.00
1B	0.99	1.00	0.99	0.99
1D	0.99	0.99	0.93	1.00
2A	0.99	0.98	0.98	1.00
2B	0.99	0.99	0.97	0.99
2D	0.99	0.97	0.91	1.00
3A	0.99	0.98	0.99	0.99
3B	0.99	0.99	0.95	0.98
3D	0.99	1.00	0.91	0.99
4A	0.98	0.98	1.00	0.99
4B	0.96	0.99	0.95	0.99
4D	1.00	0.99	1.00	1.00
5A	0.99	1.00	1.00	0.99
5B	1.00	0.99	0.99	1.00
5D	0.97	0.87	0.97	0.97
6A	0.98	0.99	0.98	0.99
6B	0.99	0.98	1.00	1.00
6D	0.97	0.79	0.95	0.99
7A	0.95	0.95	0.98	0.98
7B	0.99	0.99	0.98	0.99
7D	1.00	1.00	0.98	1.00

### Integration of the Consensus Map with the Wheat EST Chromosome Bin Map

In comparing the flanking sequences of SNPs in the consensus map with 6,596 mapped wheat ESTs on the wheat Chromosome Bin Map (Qi et al., 2004), 819 EST loci corresponding to 114 chromosome bins in the map were integrated into the genetic consensus map based on 1,221 linked SNP markers (**Table 4** and Supplementary Table S3). The distribution of the ESTs on chromosomes ranged from 4 (4D) to 74 (1B), with a mean of 39 per chromosome, and the number of assigned SNPs per chromosome ranged from 4 (4D) to 106 (1B), with a mean of 58. Coverage of each chromosome on the consensus map varied from 25% (4D) to 100% (2A, 1B, 3B, and 6A), with an average 71.7%. The D genome had the least coverage at 44.2%, whereas the A and B genomes had much higher coverages of 80 and 89.4%, respectively.

**Table 4:** Alignment of consensus map with wheat Chromosome Bin Map (Qi et al., 2004).

Chromosome	EST^∗^	SNP^∗∗^	Corresponding bins	All bins	Coverage (%)
1A	38	50	3	6	50
2A	55	76	4	4	100
3A	44	54	4	6	66.7
4A	50	80	8	9	88.9
5A	43	63	7	9	77.8
6A	56	96	6	6	100
7A	49	68	8	10	80
Genome A	335	487	40	50	80
1B	74	106	11	11	100
2B	65	100	7	8	87.5
3B	59	86	8	8	100
4B	31	41	6	7	85.7
5B	48	83	9	11	81.8
6B	52	85	5	6	83.3
7B	57	93	5	6	83.3
Genome B	386	594	51	57	89.4
1D	19	28	2	7	28.6
2D	27	39	3	6	50
3D	12	19	4	6	66.7
4D	4	4	2	8	25
5D	12	17	3	8	37.5
6D	13	22	5	10	50
7D	11	11	4	7	57.1
Genome-D	98	140	23	52	44.2
Total	819	1,221	114	159	71.7

*^∗^ Number of ESTs integrated into the consensus map. ^∗∗^ Number of SNPs assigned onto the specific chromosome bins.*

### Map Comparison

The consensus map in the present study was compared with that reported previously (Wang et al., 2014). A total 24,710 SNP markers were shared between the two maps; among them, 22,736 markers mapped to the same chromosome locations, whereas 1,974 showed different chromosome assignments (**Table 5**). The orders of the 22,736 markers in the present map were consistent with the corresponding positions in Wang et al. (2014) with Spearman rank correlation coefficients ranging from 0.84 (6D) to 0.99 (6A), and a mean correlation of 0.95. In addition to the common markers between two maps, 4,982 markers were only placed in the present consensus map.

**Table 5:** Comparison between the present consensus map and that of Wang et al. (2014).

Chromosome	Markers^##^	Bins^##^	Markers^#^	Bins^#^	Consistent markers	New mapped markers	Inconsistent markers	Person correlation coefficient
1A	2,072	303	1,496	534	1,197	223	76	0.98
1B	3,471	352	2,282	470	1,856	294	132	0.96
1D	1,024	179	774	170	679	61	34	0.92
2A	2,090	258	1,885	549	1,350	328	207	0.93
2B	3,250	387	2,872	822	2,098	553	221	0.98
2D	1,491	241	728	194	552	103	73	0.94
3A	1,800	237	1,437	476	1,104	240	93	0.97
3B	2,491	318	2,261	702	1,612	509	140	0.93
3D	883	187	410	118	250	100	60	0.88
4A	1,928	269	1,547	468	1,258	197	92	0.96
4B	1,444	248	865	342	688	138	39	0.97
4D	296	101	118	75	87	22	9	0.91
5A	2,454	338	1,371	537	1,047	289	35	0.97
5B	2,964	376	2,741	763	2,259	364	118	0.98
5D	1,004	199	391	157	231	128	32	0.92
6A	2,133	265	1,953	455	1,491	338	124	0.99
6B	2,528	278	2,022	623	1,568	336	118	0.98
6D	571	127	388	136	260	74	54	0.84
7A	2,662	304	2,010	686	1,505	346	159	0.96
7B	2,461	334	1,835	556	1,502	247	86	0.98
7D	1,250	263	306	127	142	92	72	0.96
Total	40,267	5,564	29,692	8,960	22,736	4,982	1,974	0.95

*^#^Data of the present consensus map. ^##^Data of the previous consensus map reported by Wang et al. (2014).*

**FIGURE 2:**
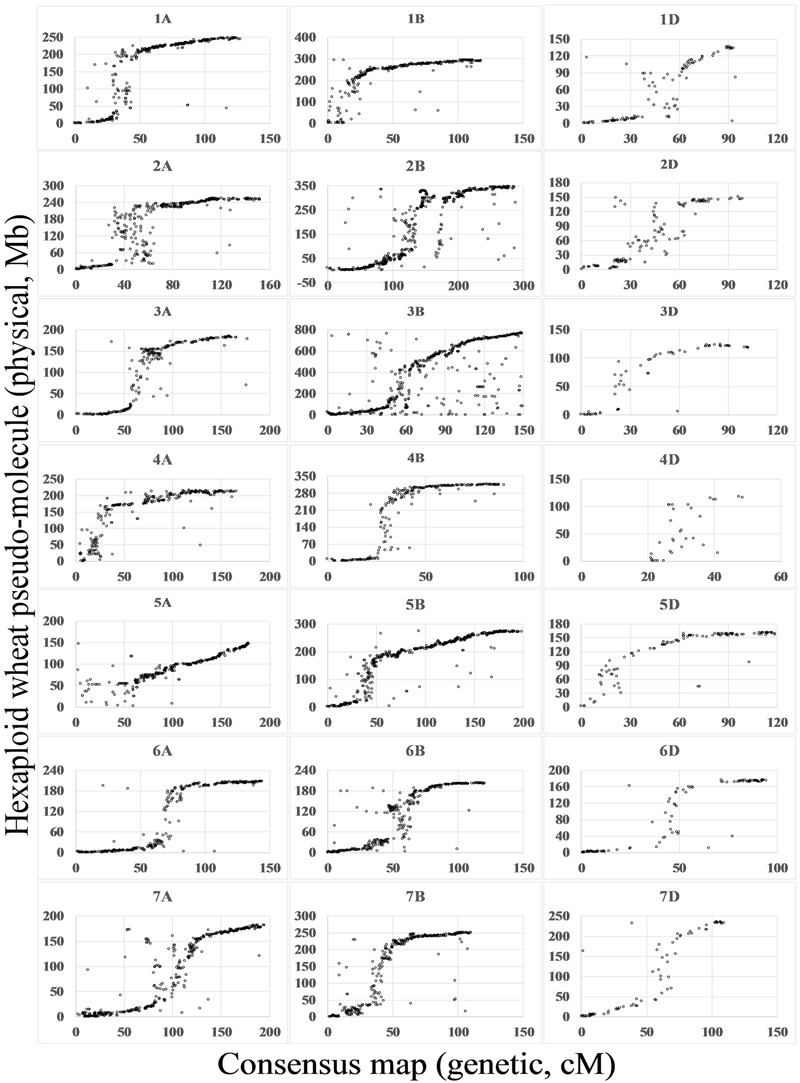
Relationship between the wheat consensus map and the physical map. Based on blastn search of flanking sequences of SNP markers, the consensus maps were aligned to the IWGSC1+popseq database (ftp://ftp.ensemblgenomes.org/pub/plants/release-31/fasta/triticum_aestivum/dna/) (Choulet et al., 2014; International Wheat Genome Sequencing Consortium, 2014; Chapman et al., 2015). The *x-*axis indicates the position on the consensus map in cM and the *y*-axis indicates the position on physical map in Mb.

By comparison between the wheat genome sequences and flanking sequences of SNPs mapped on the consensus map, 20,580 (69%) SNPs matched the wheat genome (*E*-value < 1*e* - 10). For most chromosomes, marker orders on the consensus map were consistent with the putative physical orders on the genome. Spearman rank correlation coefficients of the genetic and physical orders ranged from 0.69 (4D) to 0.97 (1A, 4B, 5B, and 6A), with an average 0.91. SNP markers anchored to pseudo-molecules were distributed close to sigmoidal patterns for all chromosomes except 1B, 4A, 4D, and 5A (**Figure 2**).

### Analysis of Structural Rearrangement and the Collinearity among Homoeologous Chromosomes

**FIGURE 3:**
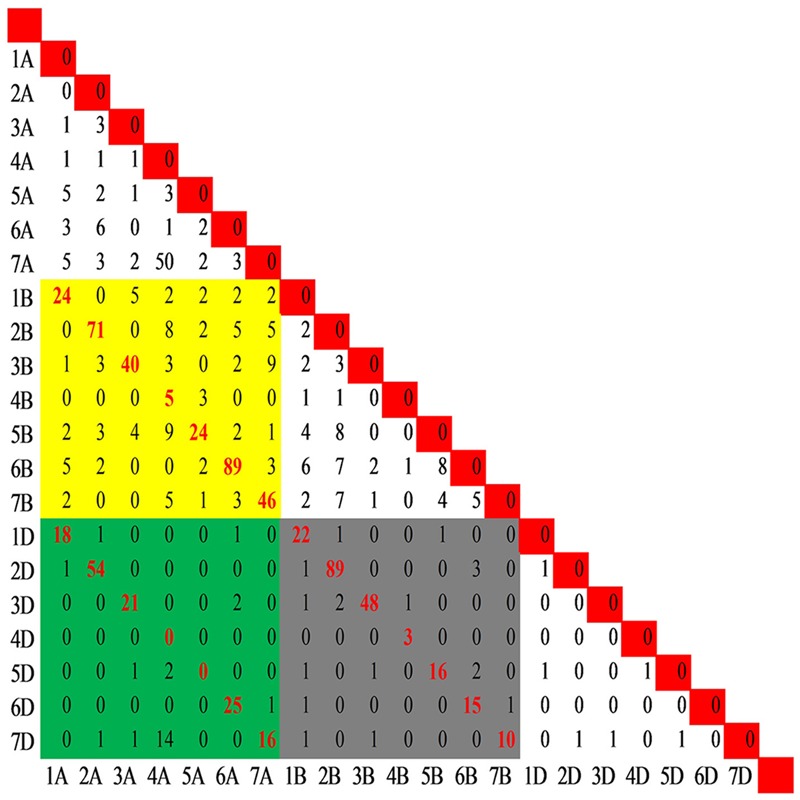
Distribution of the pair-wise SNP loci genotyped by the multi-locus SNP. A total of 942 pairs of loci including 909 pair-wise loci (e.g., 1A-1B) produced by SNPs mapped to two positions and 33 pair-wise loci (e.g., 1A-1B, 1A-1D, and 1B-1D) produced by SNPs mapped to three positions. The yellow block indicates the pairs between genomes A and B, while the green and gray blocks indicate the pairs between genomes A and D and between B and D, respectively. The red font indicates the pair-wise loci among the homoeologous chromosomes.

In the present study, 29,692 SNP loci mapped on the consensus map were genotyped by 28,761 SNP assays in the wheat 90K array. Of these, 27,841 were mapped to single positions and were designated as single-locus SNPs, whereas 920 assays mapped to more than one position and designated as multi-locus SNPs, with 909 at two positions (e.g., 1A, 1B) corresponding to 909 pair-wise loci (e.g., 1A-1B), and 11 at 3 positions corresponding to 33 pair-wise loci (e.g., 1A-1B, 1A-1D, and 1B-1D) (Supplementary Figure S3 and Table S4). Among the 942 locus pairs (**Figure 3**), 636 mapped to homoeologous chromosomes (e.g., 1A-1B), including all coupled combinations of homoeologous chromosomes except 4A-4D and 5A-5D, and the Spearman rank correlation coefficients between the duplicated loci mapped on coupled homoeologous chromosomes ranged from 0.62 (4A-4B) to 1.0 (4B-4D and 6B-6D), with an average of 0.92. Moreover, 306 pairs of loci involved in chromosomal translocations mapped to non-homoeologous chromosomes (e.g., 1A-2B or 1A-2A), with 165 being assigned intra-genomic (e.g., 1A-2A) and 141 inter-genomic (e.g., 1A-2B) (Supplementary Table S5).

The frequency of the loci involved in translocations was unbalanced among the chromosome pairs except in the D genome with six markers corresponding to 6 chromosome pairs (**Figure 3**). Fifty SNP loci in the 4A-7A pair and 14 SNPs in the 4A-7D pair suggested translocation between groups 4 and 7. In addition, translocations between chromosomes 4A and 5B was indicated by 18 non-homoeologous loci assayed by 9 SNPs. Combining the genetic positions of loci involved in translocations on the consensus map, the cyclic translocation 4AL-5AL-7BS was confirmed (Supplementary Table S5). Several other translocations were also identified (e.g., 1A-7A, 2A-6A, 4A-2B, 7A-3B, 2B-6B, and 5B-6B).

### Characteristics of the Integrated Consensus Map

An integrated consensus map constructed with the data of the present study and those from five previous reports comprised 52,607 markers belonging to 14,485 recombinant bins, including 51,655 SNP, 667 SSR, 266 DArT and 19 other markers. All markers were assigned to 21 linkage groups corresponding to 21 wheat chromosomes, and the number of markers in each chromosome ranged from 379 (4D) to 4,326 (1B) with an average of 2,505 markers per chromosome (Supplementary Table S6). A total of 9,691 markers were newly mapped compared with the previously consensus map (Wang et al., 2014). The Spearman rank correlation coefficients of the marker order between the integrated consensus map and the consensus map by Wang et al. (2014) ranged from 0.94 to 0.99. Compared with a tetraploid consensus map reported by Maccaferri et al. (2015), 25,942 markers were in common between the two maps, with 26,665 being mapped exclusively in our map (19,463 in the A and B genomes, 7,202 in D genome). Among the shared markers, 22,646 showed consistent chromosome assignments between the two maps, whereas 3,296 were assigned to different chromosomes. The Spearman rank correlation coefficients between homologous chromosomes of the two consensus maps ranged from 0.96 to 0.99. Rank order plots of data pairs from the two maps (Supplementary Figure S4) showed wide regions with suppressed recombination in the hexaploid wheat consensus map, particularly in the pericentromeric regions of chromosomes 1A, 1B, 2B, 3A, 4B, and 7A, and similar situation was observed on chromosomes 2A and 6A in the tetraploid wheat consensus map.

## Discussion

### Construction of Individual Maps

Three computer packages were employed in the present study to construct individual maps. In most previous studies, a single package was used for linkage map construction involving hundreds of markers (Röder et al., 1998; Paillard et al., 2003; Quarrie et al., 2005; Galeano et al., 2011; Talukder et al., 2014). In some other cases involving high-throughput marker technology, such as DArT and SNP arrays, packages were combined to construct linkage maps (Deokar et al., 2014; Maccaferri et al., 2014; Winfield et al., 2016). As increasing numbers of SNPs were identified in plants (Breseghello and Sorrells, 2006; Hyten et al., 2010; Wu et al., 2010; Lai et al., 2012), the challenge of integrating them into linkage maps greatly increased, and the weaknesses of packages, such as MapMaker/EXP (Lander et al., 1987), MapManager (Manly et al., 2001), JoinMap4.0 (Stam, 1993), QTL IciMapping V4.0 (Li et al., 2007), MSTMAP (Wu et al., 2008a) and MapDisto (Lorieux, 2012), became apparent when marker numbers reached 10,000 to 100,000, MapMaker/EXP (Lander et al., 1987) and MapManager (Manly et al., 2001) were quite efficient during the era of SSR markers, but these became powerless when thousands of markers needed to be processed; JoinMap 4.0 (Stam, 1993) had a convenient grouping function which tested LOD scores in the range 3 to 100, but the speed of the ordering was unsatisfying, taking more than 1 week to group about 200 markers; QTL IciMapping V4.0 (Li et al., 2007) had the powerful Bin function useful in removing redundant markers, but poor in grouping function and had to test LOD values successively in trying to group markers. MSTMAP (Wu et al., 2008a) was good at managing thousands of markers, but sensitive to data quality and loss in the flexible grouping function; MapDisto (Lorieux, 2012) had a friendly interface and was powerful in handling thousands of markers, but poor in Bin function and weak in the grouping function. Features such as fast ordering algorithms, flexible functions for reducing the complexity of calculation, and separating groups are important to process large numbers of markers, but most of the softwares do not possess all these characteristics. In the present study, over 10,000 markers were mapped in each population; we used QTL IciMapping V4.0 (Li et al., 2007) for binning redundant markers, JoinMap 4.0 (Stam, 1993) for efficient grouping and MapDisto (Lorieux, 2012) for a fast ordering algorithm in developing the four individual maps.

### Construction and Features of the Integrated Consensus Map

The basis of map mergence was a large number of common markers across the different populations. Four individual populations were used to construct the consensus map; details of common markers in two, three and all four maps are shown in **Figure 1**.

Several programs can be used to develop the consensus map, such as JoinMap (Stam, 1993), Multipoint (Ronin et al., 2012), MergeMap (Wu et al., 2008b) and LPmerge (Endelman and Plomion, 2014). Considering the drawbacks of some programs, such as the computer time required for JoinMap and Multipoint, the other two softwares were trialed for construction of the consensus map. Because the integration maps constructed by LPmerge were dumbbell-like, with most markers concentrated at ends of chromosomes, MergeMap (Wu et al., 2008b) was finally chosen for construction of the consensus map. This software has been used frequently in other crop plants (Close et al., 2009; Galeano et al., 2011; Khan et al., 2012; Wang et al., 2014).

The consensus map from four Chinese wheat populations had a marker density of 10.21 markers/cM, much higher than that observed in the individual maps, which ranged from 3.32 markers/cM (Linmai 2 × Zhong 892) to 6.49 markers/cM (Zhou 8425B × Chinese Spring). Due to large genetic gaps among some markers, some chromosomes were split into multiple linkage groups causing discrepancies between numbers of linkage groups and chromosomes in two populations (Linmai 2 × Zhong 892, Gaocheng 8901 × Zhoumai 16). The gaps were filled by markers located in corresponding regions in the other maps. Overlapping regions between individual maps were enriched by additional markers, and gaps of over 10 cM were not observed in the consensus map (Supplementary Figure S2). A high consistency of marker order between the consensus map and individual maps was confirmed by pairwise Spearman rank correlation coefficients that evaluated the degree of marker order correspondence (**Table 3**). A few discrepancies in the order of common markers between individual maps reflected differences in genomes between individual populations or were errors in the specific individual maps, and were corrected. Hence a more reliable marker order was obtained with the consensus map. The percentages of the three sub-genome lengths (A = 39.2%, B = 37.2%, D = 23.6%) in the present consensus map were closer to estimates from somatic metaphase chromosome size (A = 34%, B = 37.2%, D = 28.8%) given by Gill et al. (1991), compared with those (A = 34.1%, B = 31%, D = 34.9%) in Wang et al. (2014).

Previously, 40,267 SNP markers were mapped on a consensus map using the wheat 90K iSelect array (Wang et al., 2014); detailed chromosomal locations are important for use of SNP markers in the MAS, QTL mapping and gene cloning. However, around half of the SNP markers in the 90K array are not mapped, and it is necessary to map them and validate their positions. Compared with the consensus map constructed by Wang et al. (2014), 4,982 SNP markers were exclusively mapped in the present consensus map from four populations, and chromosome assignments of 22,736 SNP markers were confirmed. This represents a significant contribution for genetic studies using SNP markers from the wheat 90K iSelect assay.

By comparison with the genome sequence (**Figure 2**), most of the linkage groups agreed with structural partitioning as described for chromosome 3B (Choulet et al., 2014). The sigmoidal patterns correspond to two distal regions of the chromosome and a middle region including the centromeric and pericentromeric regions. With only 69% of SNP flanking sequences aligning to the draft sequence, several linkage groups showed incomplete chromosomal structures, such as the near-deficient short arms of 1B, 4A, 4D, and 5A. Due to the enrichment of repeat sequences in the centromeric and pericentromeric regions and the lower sequencing depth regions of the draft sequence (Choulet et al., 2014), marker orders in the centromeric and pericentromeric regions were chaotic, particularly in chromosomes such as 2A and 6B.

Due to the convenience and accuracy of high-throughput SNP arrays, SNP markers are widely used in the wheat genetic research, and a reliable reference map is of high importance for genetic studies, such as the milestone of wheat SSR consensus map (Somers et al., 2004) making an important contribution in the era of SSR markers. Nevertheless, there is only one public consensus map based on wheat 90K SNP array for hexaploid wheat (Wang et al., 2014). In the present study, we constructed an integrated 90K SNP consensus map using four mapping populations derived from Chinese wheat lines, six used by Wang et al. (2014), seven from Cavanagh et al. (2013), and three from other studies (Cabral et al., 2014; Gardner et al., 2016; Zhai et al., 2016). Finally, an integrated consensus map combining polymorphic information from 20 populations were developed, comprising 52,607 markers. In addition to 51,655 SNPs, 667 SSR, 266 DArT, and 19 other markers were also integrated in the map.

### Detection of Collinearity and Chromosomal Rearrangements by the Consensus Map

The evolutionary history of common wheat suggested that collinearity between A and B genomes was better than that with the D genome (Dubcovsky and Dvorak, 2007); it was also evident in the present study that the coupled loci derived from multi-locus SNPs mapped to chromosome pairs corresponding to the A and B genomes more so than those mapped to B–D and A–D chromosomes, and synteny of loci among sub-genomes was confirmed in the consensus map (Supplementary Figures S3, S5).

Several chromosomes of hexaploid wheat contain translocations of considerable size, and the evolutionary evidence for translocations involved in chromosome arms 4AL, 5AL, and 7BS has been firmly established (Liu et al., 1992; Devos et al., 1995; Ma et al., 2013). In our consensus map, 50 coupled loci derived from multi-locus SNPs were identified in the 4A-7A pair, 14 corresponded to 4A-7D, and 9 to 4A-5B. Considering the genetic positions of the loci (Supplementary Table S5) these translocations trace to the cyclic 4AL-5AL-7BS translocation. Nearly half of the putative translocations in the present study are inter-genomic, supporting the conclusion that recombination between homoeologous chromosomes was a common event (Wendel and Wessler, 2000).

### Implications of the Integrated Consensus Map

The present consensus map in common wheat increases the marker density and genome coverage compared to maps developed from single bi-parental populations, and makes it possible to compare the locations of QTL related to the important phenotypic traits among different bi-parent populations. The limited marker density present in regions with QTL identified in individual bi-parental populations can be resolved by the large number markers in the regions of interest on the consensus map. The consensus map providing high-density markers along the chromosomes represents a significant advantage for fine mapping of QTL, and transfer of QTL among different genomic backgrounds. As association mapping has been performed for many traits in common wheat (Tommasini et al., 2007; Kollers et al., 2013; Zanke et al., 2014), the consensus map could be useful for further association analyses, facilitating estimation of linkage disequilibria and QTL detection by traditional interval mapping (Neumann et al., 2011). Consensus maps can be used to perform meta-QTL analysis (Mace and Jordan, 2011; Quraishi et al., 2011) in combination with genetic marker and QTL data on a single map. The present integrated consensus map has 52,607 markers covering all the hexaploid wheat chromosomes, and the integrated SSR and DArT markers provides useful information for comparison with previous research. The SNP markers mapped on the map are gene-derived, and will facilitate association mapping, meta-QTL analysis and positional cloning, and will have application in wheat breeding.

## Ethics Statement

We declare that these experiments comply with the ethical standards in China.

## Author Contributions

WW, FG, JL, HJ, and SZ performed the experiment. XX, YQ, and ZH designed the experiment. WW, XX, and ZH wrote the paper.

## Conflict of Interest Statement

The authors declare that the research was conducted in the absence of any commercial or financial relationships that could be construed as a potential conflict of interest.

## Footnotes

1 http://www.illumina.com/

2 http://cn.capitalbio.com/

3 https://urgi.versailles.inra.fr/download/iwgsc/Survey_sequence/

4 http://wheat.pw.usda.gov/blast/blast_databases.html

5 ftp://ftp.ensemblgenomes.org/pub/plants/release-31/fasta/triticum_aestivum/dna/
