# Supplementary material for: A High-Density Consensus Map of Common Wheat Integrating Four Mapping Populations Scanned by the 90K SNP Array
Source: Front Plant Sci. 2017 Aug 9;8:1389. doi: 10.3389/fpls.2017.01389 (PMC5552701; doi:10.3389/fpls.2017.01389)
Supplement: Supplementary file 7 [file Image_1.PDF]

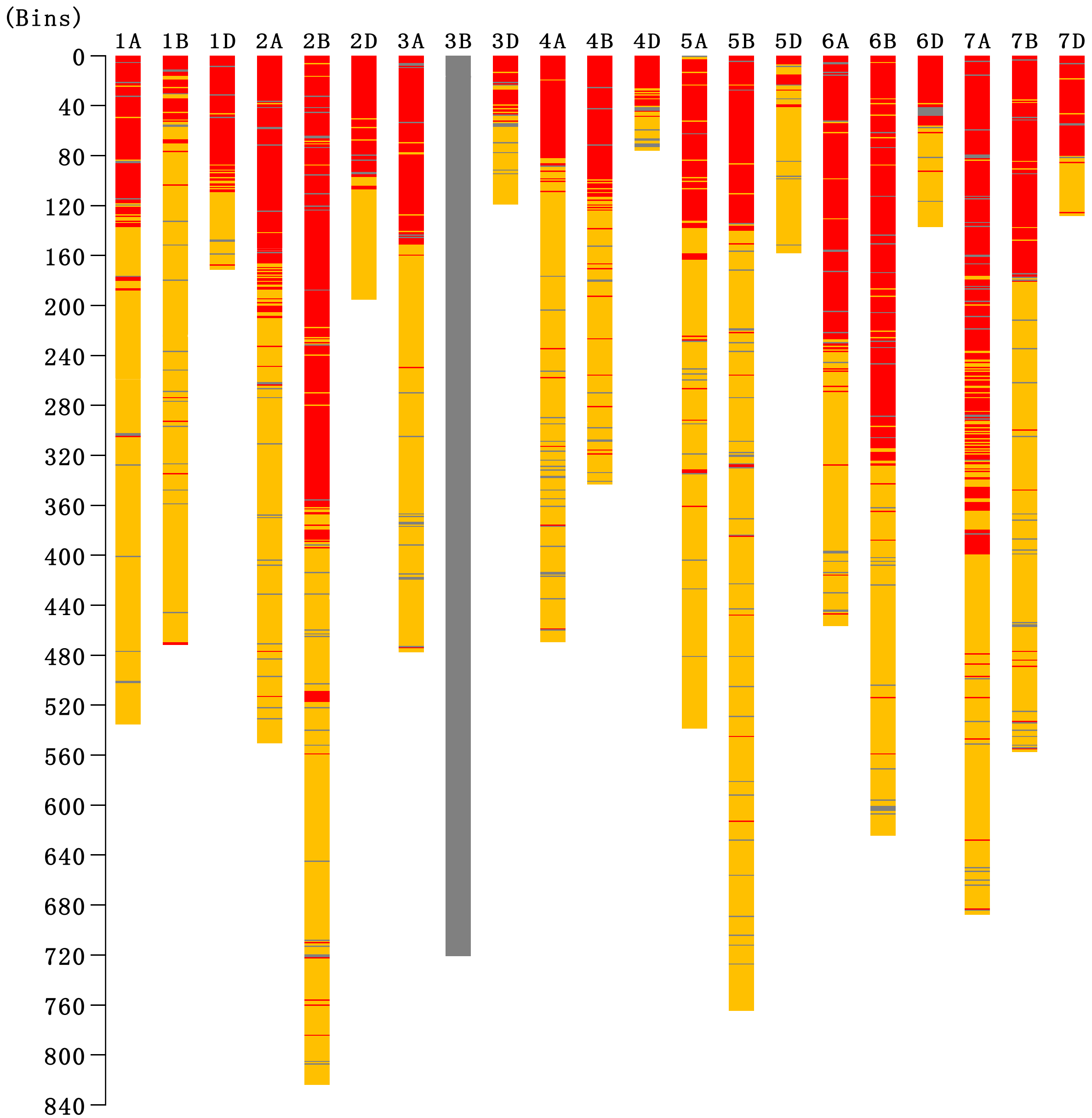

Figure S1: Assignment of linkage groups to wheat chromosomes.

The red and orange colors represent the short and long arms, respectively; gray color undetermined arm locations.
