# Supplementary material for: A High-Density Consensus Map of Common Wheat Integrating Four Mapping Populations Scanned by the 90K SNP Array
Source: Front Plant Sci. 2017 Aug 9;8:1389. doi: 10.3389/fpls.2017.01389 (PMC5552701; doi:10.3389/fpls.2017.01389)
Supplement: Supplementary file 8 [file Image_2.PDF]

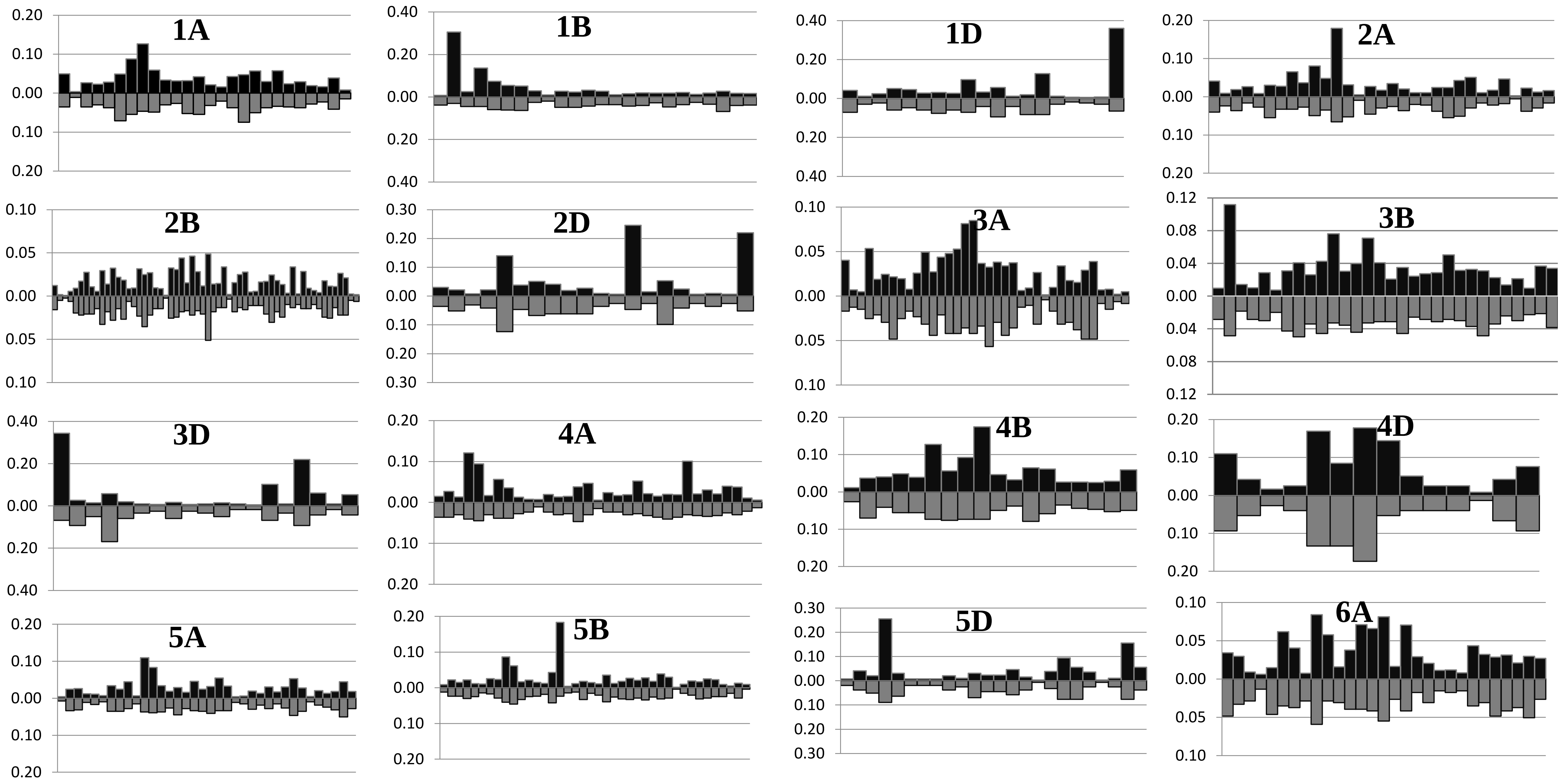
1A
0.20
0.10
0.00
0.10
0.20
1B
0.40
0.20
0.00
0.20
0.40
1D
0.40
0.20
0.00
0.20
0.40
2A
0.20
0.10
0.00
0.10
0.20
2B
0.10
0.05
0.00
0.05
0.10
2D
0.30
0.20
0.10
0.00
0.10
0.20
0.30
3A
0.10
0.05
0.00
0.05
0.10
3B
0.12
0.08
0.04
0.00
0.04
0.08
0.12
3D
0.40
0.20
0.00
0.20
0.40
4A
0.20
0.10
0.00
0.10
0.20
4B
0.20
0.10
0.00
0.10
0.20
4D
0.20
0.10
0.00
0.10
0.20
5A
0.20
0.10
0.00
0.10
0.20
5B
0.20
0.10
0.00
0.10
0.20
5D
0.30
0.20
0.10
0.00
0.10
0.20
0.30
6A
0.10
0.05
0.00
0.05
0.10

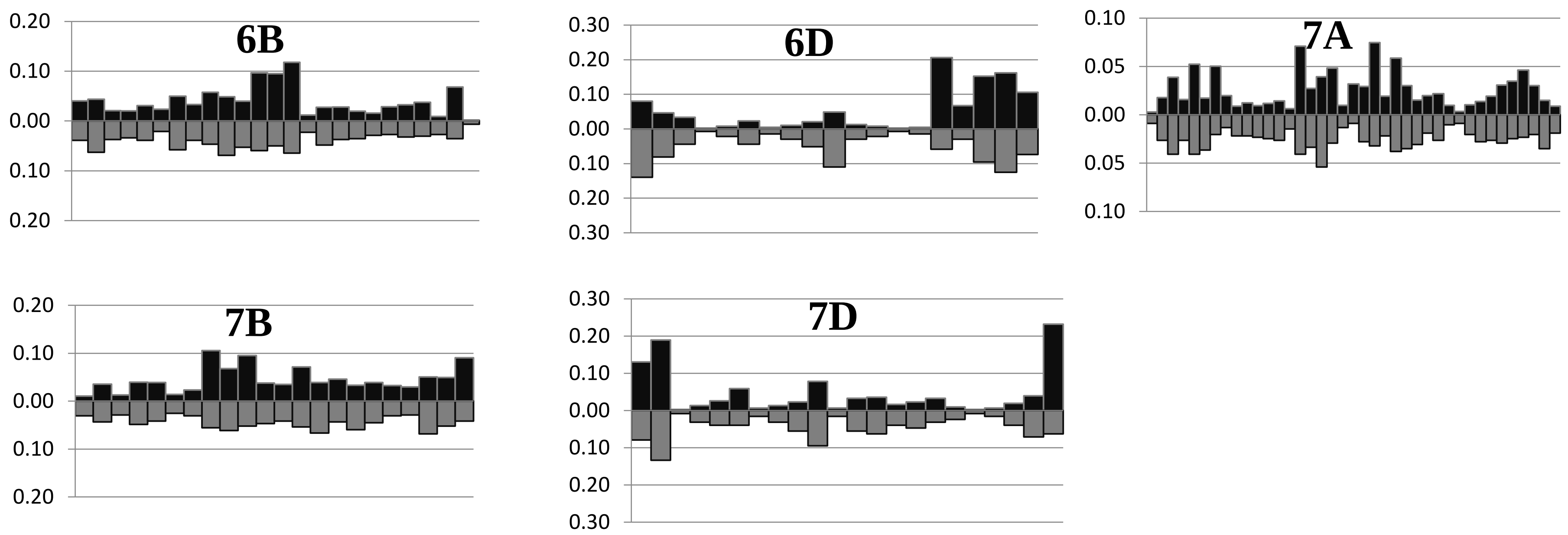

Figure S2: Distribution of SNPs on the consensus map.

Upper, proportion of markers based on 5 cM relative to total markers mapped on chromosome: lower, proportion of bin markers based on 5 cM relative to total bins on the chromosome.
