# Supplementary material for: A High-Density Consensus Map of Common Wheat Integrating Four Mapping Populations Scanned by the 90K SNP Array
Source: Front Plant Sci. 2017 Aug 9;8:1389. doi: 10.3389/fpls.2017.01389 (PMC5552701; doi:10.3389/fpls.2017.01389)
Supplement: Supplementary file 9 [file Image_3.PDF]

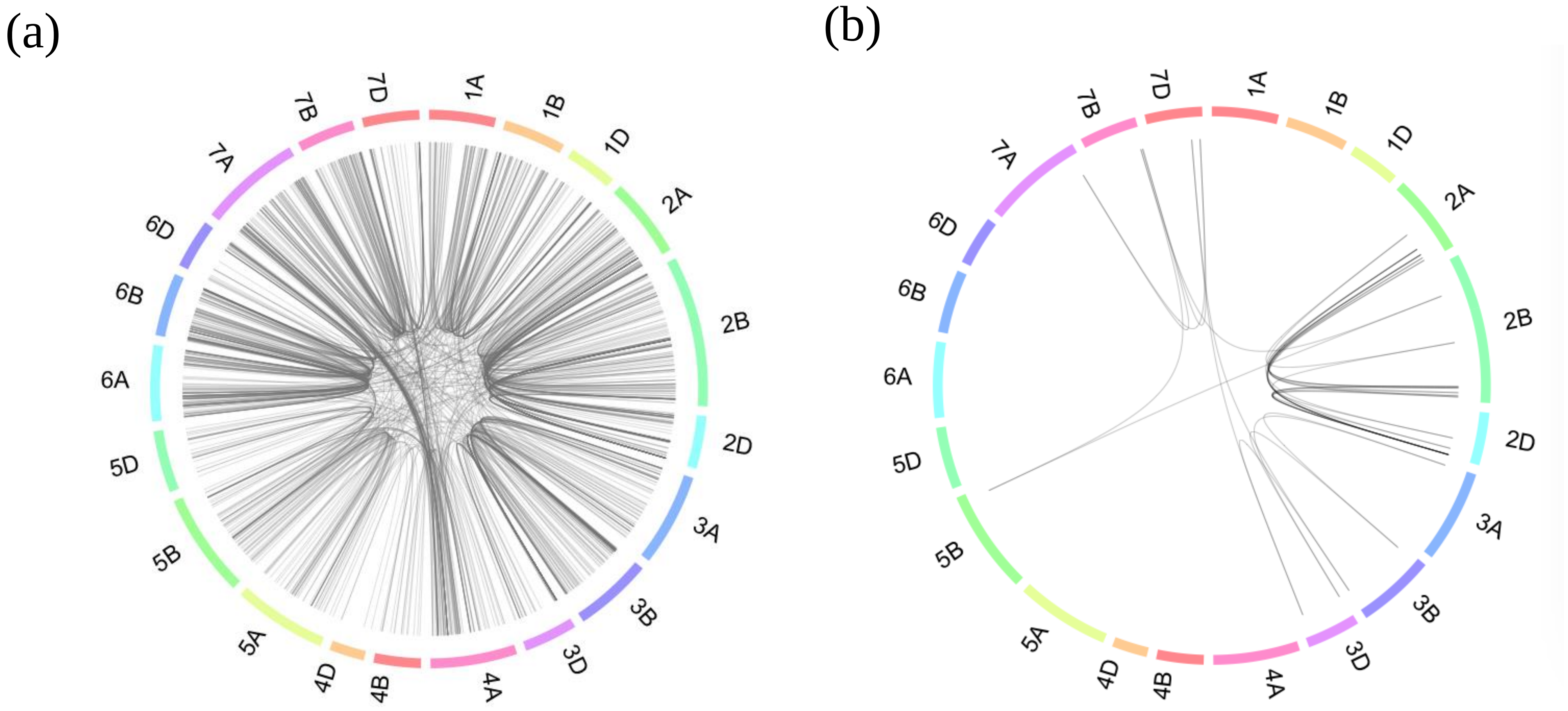

Figure S3: Comparative analysis of the orders of SNP loci based on the multi-locus SNPs.

Each of 920 SNPs mapped to more than one position named as multi-locus SNPs; of these, 909 mapped at two positions (a); and 11 multi-locus SNPs were detected at three positions (b).
