# Supplementary material for: A High-Density Consensus Map of Common Wheat Integrating Four Mapping Populations Scanned by the 90K SNP Array
Source: Front Plant Sci. 2017 Aug 9;8:1389. doi: 10.3389/fpls.2017.01389 (PMC5552701; doi:10.3389/fpls.2017.01389)
Supplement: Supplementary file 10 [file Image_4.PDF]

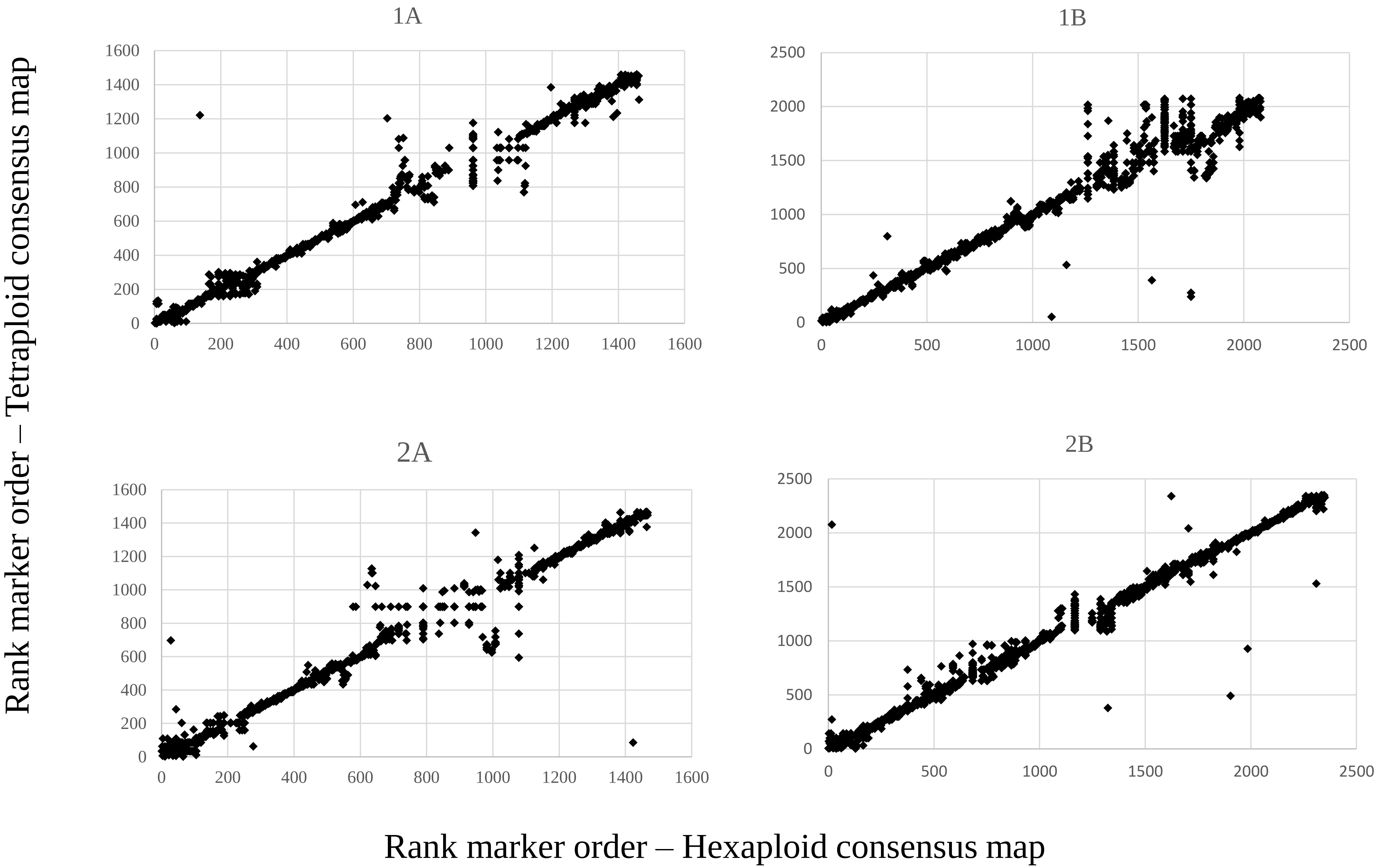
1A
1B
2A
2B
Rank marker order – Tetraploid consensus map
Rank marker order – Hexaploid consensus map

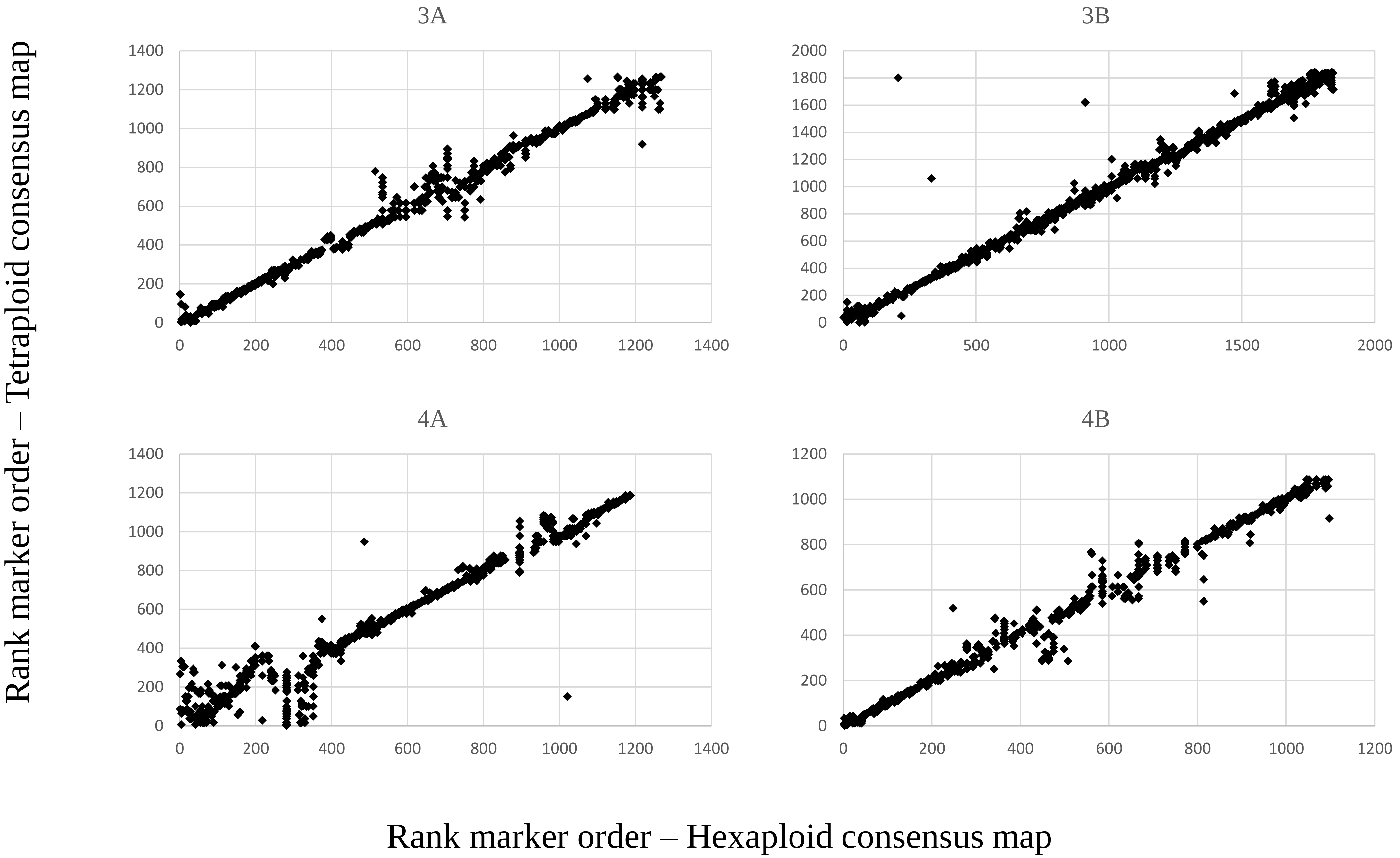

3A
3B
4A
4B
Rank marker order – Tetraploid consensus map
Rank marker order – Hexaploid consensus map

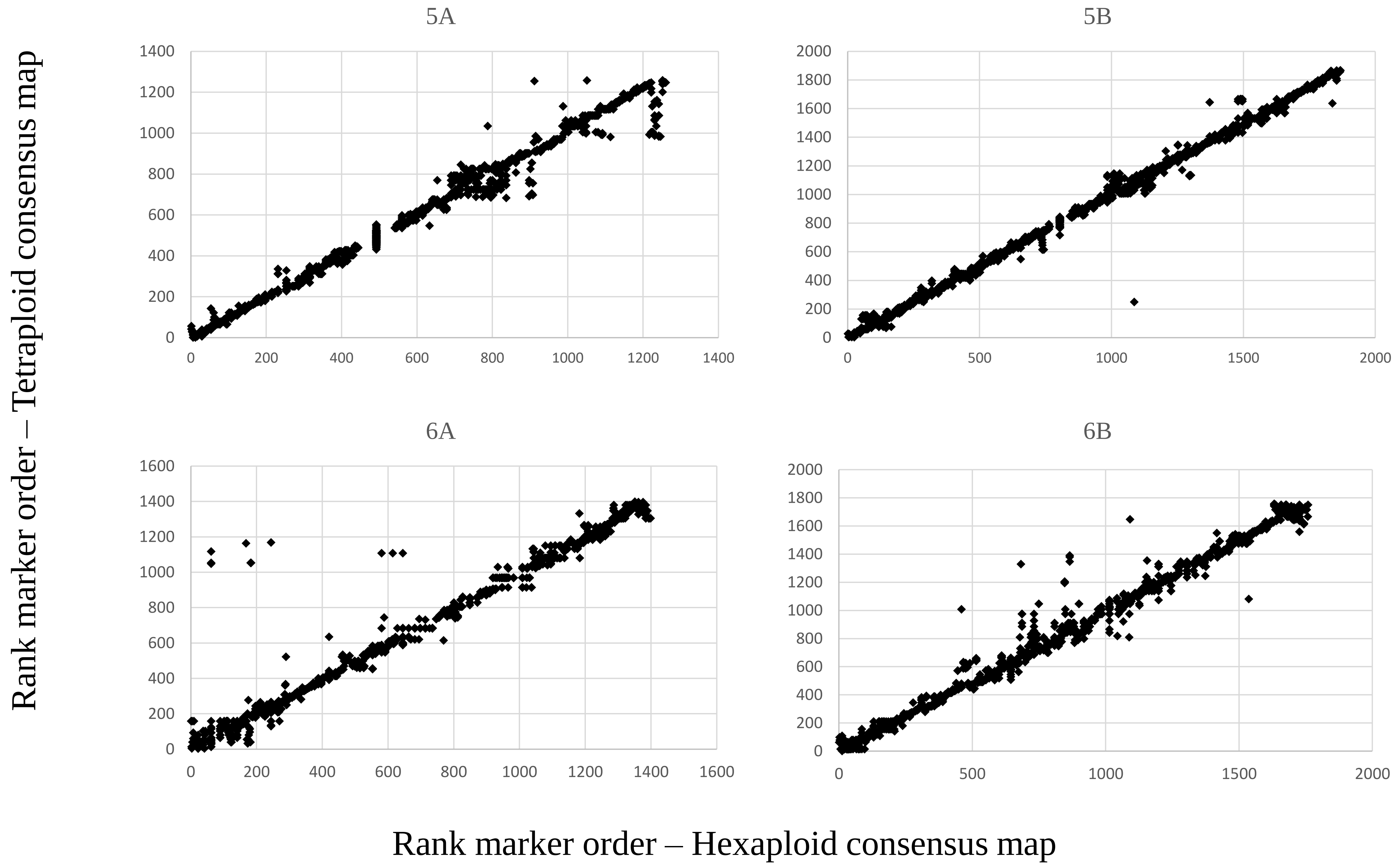

5A
1400
1200
1000
800
600
400
200
0
0
200
400
600
800
1000
1200
1400
5B
2000
1800
1600
1400
1200
1000
800
600
400
200
0
0
500
1000
1500
2000
6A
1600
1400
1200
1000
800
600
400
200
0
0
200
400
600
800
1000
1200
1400
1600
6B
2000
1800
1600
1400
1200
1000
800
600
400
200
0
0
500
1000
1500
2000
Rank marker order – Tetraploid consensus map
Rank marker order – Hexaploid consensus map

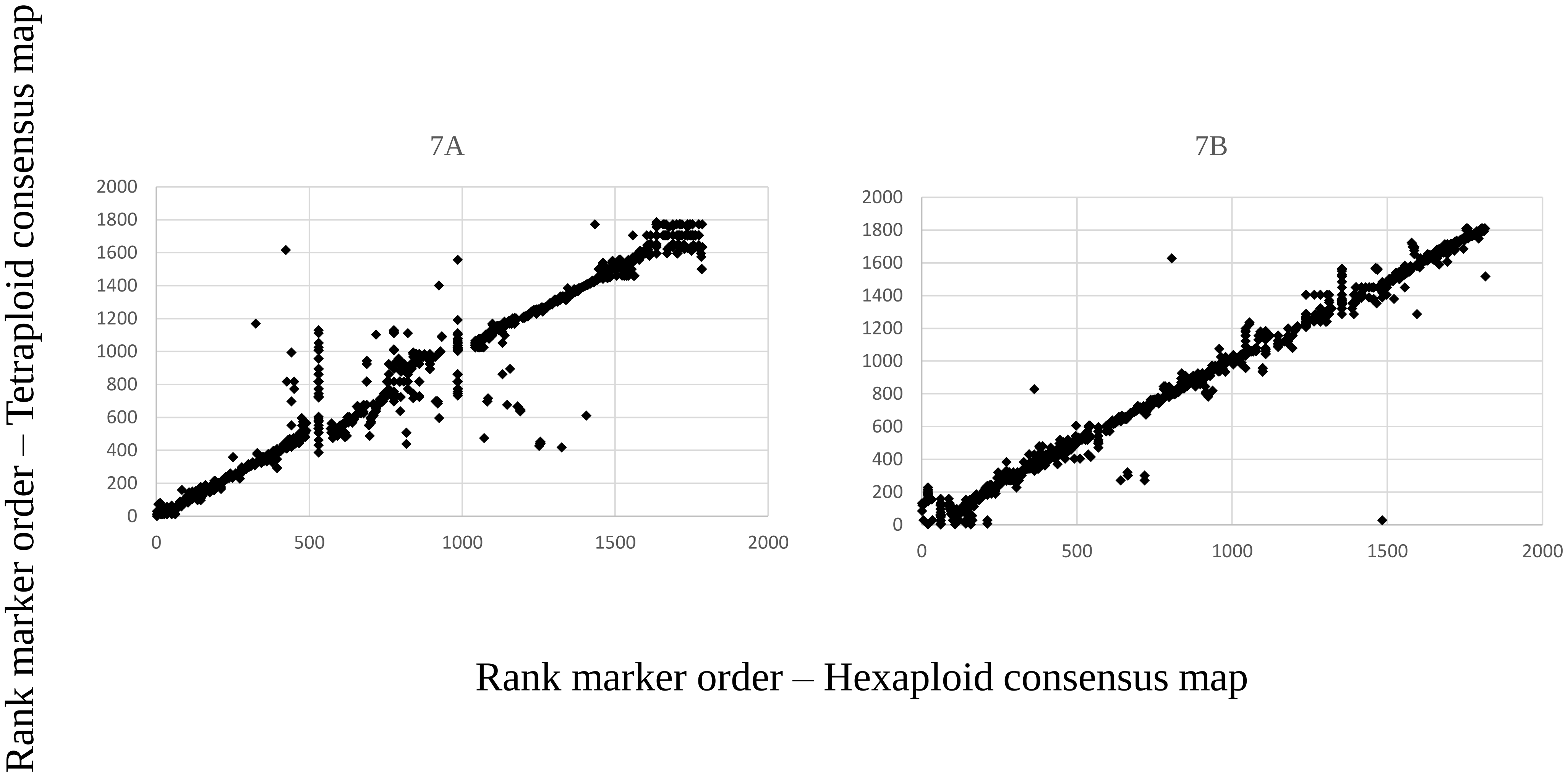

Figure S4: Rank order plots of the integrated hexaploid wheat consensus map vs. the tetraploid wheat consensus map (Maccaferri et al., 2015).

Rank order plots showed the relationship of marker order between the hexaploid and tetraploid consensus map for each of the 14 tetraploid wheat chromosomes. The *x*-axis indicates the rank marker order of the hexaploid consensus map and the *y*-axis indicates the rank marker order of the tetraploid wheat consensus map.
