# Supplementary material for: A High-Density Consensus Map of Common Wheat Integrating Four Mapping Populations Scanned by the 90K SNP Array
Source: Front Plant Sci. 2017 Aug 9;8:1389. doi: 10.3389/fpls.2017.01389 (PMC5552701; doi:10.3389/fpls.2017.01389)
Supplement: Supplementary file 11 [file Image_5.PDF]

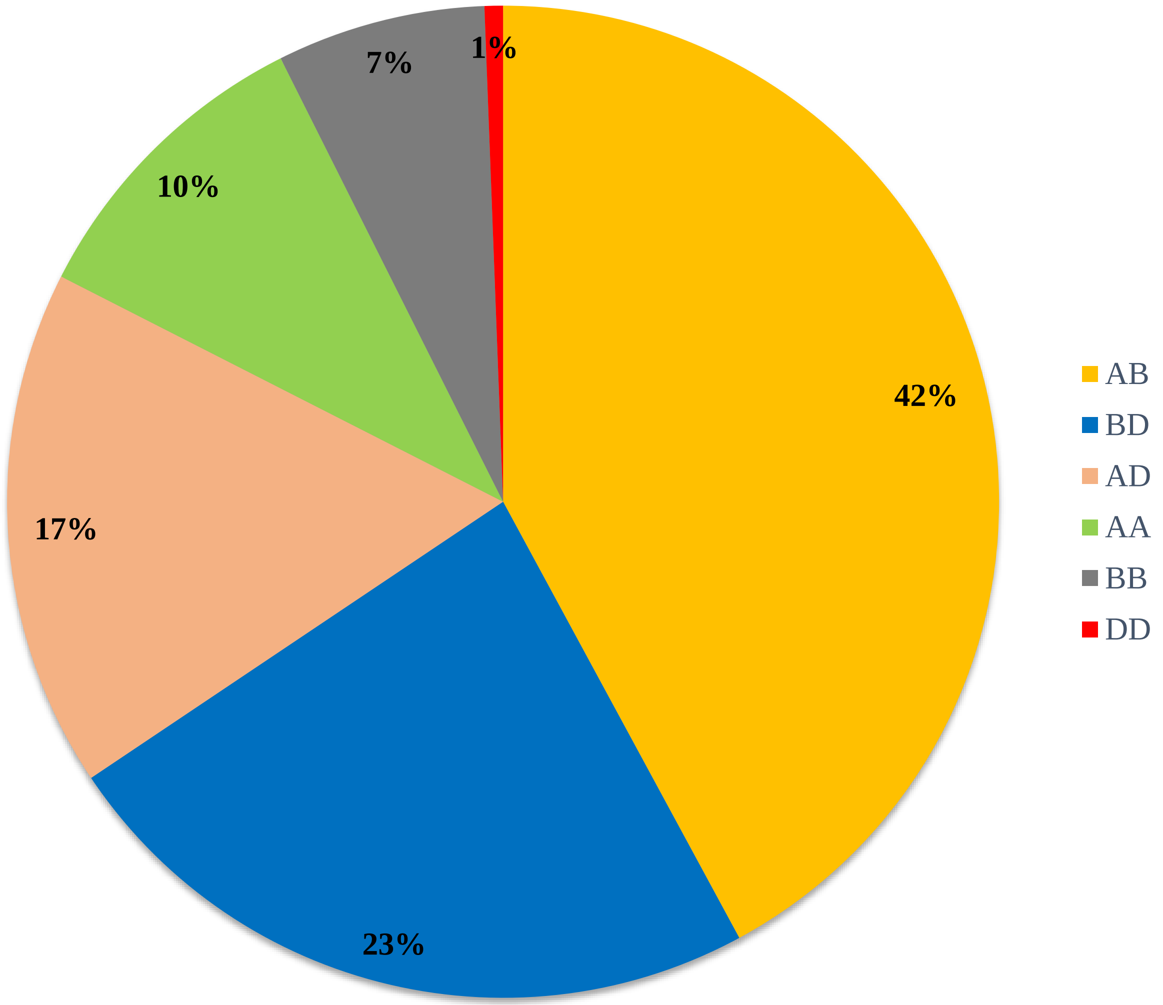


**Figure S5: Distribution pair-wise SNP loci genotyped by multi-locus SNPs**

Duplication of SNP markers in homoeologous chromosomes was more common than than in non-homoeologous chromosomes, and the relationship between genomes A and B was closer than that between genomes B and D or A and D.
